# The case for stronger regulation of private practitioners to control tuberculosis in low- and middle-income countries

**DOI:** 10.1186/s13104-015-1586-x

**Published:** 2015-10-23

**Authors:** Yodi Mahendradhata

**Affiliations:** Center for Health Policy and Management, Faculty of Medicine, Gadjah Mada University, Sekip Utara, Yogyakarta, 55281 Indonesia; Faculty of Medicine, Institute of Public Health, University of Heidelberg, Heidelberg, Germany

## Abstract

Tuberculosis case management practices of private practitioners in low- and middle-income countries are commonly not in compliance with treatment guidelines, thus increasing the risk of drug resistance. National Tuberculosis control programs have long been encouraged to collaborate with private providers to improve compliance, but there is no example yet of a sustained, large scale collaborations with private practitioners in these settings. Regulations have long been realized as a potential response to poor quality care, however there has been a lack of interest from the international actors to invest in stronger regulation of private providers in these countries due to limited evidence and many implementation challenges. Regulatory strategies have now evolved beyond the costly conventional form of command and control. These new strategies need to be tested for addressing the challenge of poor quality care among private providers. Multilateral and bilateral funding agencies committed to tuberculosis control need to invest in facilitating strengthening government’s capacity to effectively regulate private providers.

## Background

With an estimated 9.0 million incident cases in 2013, tuberculosis (TB) remains a major global health problem [1]. Most of the estimated number of cases occurred in low- and middle-income countries (LMICs) in Asia and the African Region. The six countries that stand out as having the largest number of incident cases in 2013 were India, China, Nigeria, Pakistan, Indonesia and South Africa [1]. Ironically, evidence is growing that many private providers in these LMICs are putting populations at risk by delivering poor quality TB care [2–5].

Many symptomatic TB patients in LMICs, including the poor, seek care in the private sector. Studies suggest that 80 % of the first-contact health care and nearly 50 % of TB care in India occurs in the private sector [6]. A national survey on health-seeking behavior among presumptive TB cases in Indonesia showed that 54 % first sought treatment with a private practitioner (PP) [7]. Transportation and other non-medical expenses appear to raise barriers to seeking government health facilities. Private providers in LMICs have a higher probability of being the closest care provider as they are more frequent in the community [8]. Another important reason which has also been shown to be an important reason for patients’ preferences for PPs is perceived high technical skills of the personnel [9].

Private providers however remain largely unregulated, with no effective policy or legislation to monitor them [10]. Consequently, TB diagnosis and treatment practices in the private sector is commonly not in line with current national and international guidelines [11, 12]. In Pune, India, despite a decade of training, high proportions of private providers resorted to TB serology for diagnosis [13]. A recent study in Andhra Pradesh, India reported that only one-third of PPs prescribed the correct tuberculosis TB treatment regimen while two-third prescribed a large variety of different prescriptions [14]. They also reported that many PPs prescribed second line anti TB drugs in contrary to international recommendation. The widespread use of second line anti-TB drugs in the private sector in India, Pakistan and Indonesia has been well documented by a recent study [15]. Similar patterns of insufficient knowledge and poor quality care among private providers have also been reported for other diseases of public health importance, such as malaria [4, 16–18]. Poor TB case management practices are well known to increase the risk of drug resistance [19]. Thus, these disturbing evidences of inappropriate TB case management among private providers in LMIC warrants considerable alarm for the global health community.

Collaboration with private providers have been advocated as a key element of disease control strategy in LMICs [20–22]. Accordingly, the Public–Private Mix (PPM) approach have long been incorporated as a key component of the WHO’s Stop TB Strategy [23]. The Technical Review Panel of the Global Fund to fight AIDS, TB and Malaria also considers PPM as an important attribute when assessing the technical quality of a proposal [24]. PPM in TB control thus has often been cited as examples of successful private provider engagement. There is actually no example yet however of large-scale, successful, sustained collaboration with private care providers for TB control [11, 25]. PPM have been demonstrated to be feasible in the cities of Pakistan, but with high cost [10]. Attempts to scale up PPM in 14 cities in India yielded varied results and revealed considerable challenges as well as barriers [24]. Despite PPM efforts for many years, many PPs in India still could not prescribe correct TB treatment regimens [26]. It is seemingly unrealistic to expect National TB control Programmes (NTP) to be able to collaborate with thousands of PPs adequately and sustainably in high burden settings [26]. Furthermore, the reality is many private providers in these countries are not interested in partnering with NTPs [11, 24, 27].

Regulation is a key element, which has been practically neglected in previous efforts by disease control programs to address the challenges raised by private practitioners. Ironically, regulation actually has long been recognized as a potential response to address the many problems which arise in the private delivery of health services [28]. It is seen as having a crucial balancing role in the context of uncontrolled growth of the private sector. Studies in India revealed that factors that contribute to the poor quality of services offered by the private sector are a lack of monitoring by authorities, outdated and inadequate legislation, and the inability or failure of the government to enforce existing regulations [29]. Poor regulation has also been identified as one of the key barriers for successful engagement of private providers in TB control [11, 30]. The neglect of regulation by NTPs in previous strategies to deal with private providers however seemingly has been based on the argument that regulations are easier to enact than enforce in the current context of weak health systems in LMICs and that regulation cannot be a panacea to guarantee adherence to rules by all care providers [31, 32]. There are elements of truth in such argument.

## The challenges of regulating PPs’ case management practices in LMICs

NTP have little power over PPs in LMICs as quality of healthcare provision in these settings is essentially addressed through the self-regulating function of medical professionals [33]. Some middle-income countries are now also starting to use accreditation as a means to improve quality and standards of facilities. Despite the existence of these basic regulatory legislation in most LMICs, the degree to which regulations are enforced is low [28]. Regulation enforcement in Zimbabwe has been reported to be mired by weaknesses of the main regulatory body (i.e. the Health Professions Council), inadequate design of current regulation and insufficient resources [34]. India’s formal approach of enforcing regulations through administrative and bureaucratic controls does not appear to be working well, despite having established a comprehensive formal legal system that provides a minimum of protection to the public [35]. The respective medical councils in states in India have been reported to not be enforcing the laws relating to the registration and licensing of individual practitioners [29]. The gap in effective regulation in India has also been exacerbated by the interests of the health professions, which have not focused on the key regulatory concerns [28]. In China, key challenges of health care regulation include weaknesses in the regulatory capacity of local governments, their accountability and the limited voice for consumers through the media or civil society [36].

Each country evidently poses specific challenges for effective health care regulation. Some general patterns however can be observed. Institutional capacity for enforcing regulation for instance is generally weaker in low income countries (LICs) than in middle income countries (MICs) [37]. MICs may have more resources to channel into regulation. In Thailand, roles and responsibilities of regulators were found to be comprehensive and administrative structures, rules, incentives as well as standards were in place [37]. This suggests that Thailand has reached a stage of institutional capacity which has not been achieved in LICs, mostly due to the lack of institutional development and the limited availability of resources.

The findings above however do not justify throwing out the baby with the bath water. First, it is not realistic to expect to have effective health care regulation systems in place within a short period. Even in affluent settings such as US, it took generations for regulatory bodies such as the Food and Drug Administration to achieve its status as an internationally respected, science-driven regulator, and still they are not without problems [38]. Despite the complexity and the problems of the health care regulation system in the US, it remains to be considered essential for the health care system to function well [39]. Second, it is impossible to implement well any regulatory strategy with inadequate resources. Regulation is clearly not costless, requiring skilled personnel and unit with adequate resources for data collection, inspection and monitoring [37]. The introduction of accreditation as a quality control measure in India for instance received widespread support from both public sector regulators and private hospitals, but implementation was eventually hampered by lack of resources to finance the accreditation system [37]. Third, most of the challenges revolved around traditional methods of implementing regulations include mechanisms for monitoring, inducing compliance, and penalizing non-compliance [40]. These traditional forms have a long-established history in the industrialized countries and are highly resource intensive. Consequently they have been difficult for LMICs to implement. Less cumbersome and expensive regulatory approaches have been developed in recent years [40]. Hence, instead of dismissing regulation outright, the way forward is to consider these non-conventional approaches and strategies that may be more appropriate and more effective for LMICs.

## Potential nonconventional regulatory approaches and strategies

Non-conventional approaches may provide alternative means for effectively addressing the issue of poor quality care among PPs in LMICs. Mok and colleagues highlighted two strategies which are considered to be low-cost, yet potentially effective: written notices and public disclosure [40]. In the case of PPs, written notices of non-compliance to standards offer a potentially cost-effective way to enforce regulations. Warning letters do not require any official collection of data by the regulatory body as they do not carry any force of law. The PP can discuss with the regulatory body to resolve the issue if he feels that there are justifications for non-compliance. The warning letter simply serves as notice that the regulatory body believes there is non-compliance and that tougher actions could follow if the PP does not change its behavior. Such written notices have been reported to be a particularly effective way to induce compliance with regulations and standards, especially when used in conjunction with public disclosure [40]. Public disclosure itself has been reported to be an efficient way of enforcing compliance with regulations. Regulatory bodies for instance can release information on PP’s poor case management practice to the public. The threat of public pressure has been reported to be effective in achieving regulatory goals without coercion [40].

**Fig. 1:**
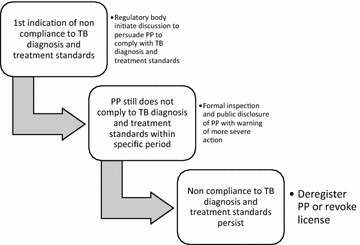
Cascade of options for regulating private practitioners’ compliance to tuberculosis (TB) diagnosis and treatment standards (adapted from Healey and Braithwaite [41] and Mok et al. [40])

Healy and Braithwaite proposed a responsive regulation approach, in which regulators employ a series of mechanisms that are responsive to the context, conduct and culture of those being regulated [41]. The core of this approach is a pyramid of regulatory mechanisms that range upwards, encompassing voluntarism, market mechanism, self-regulation, metaregulation, command and control [41]. Thus, in the case of substandard case management among PPs, regulators could use the pyramid to think responsively in choosing among a cascade of regulatory options (Fig. 1). Mok and colleagues also proposed a similar concept, refering it to as a cascading hierarchy of sanctions [40].

Black suggested the concept of de-centered regulation, which expands regulatory activities concept to actors beyond the state and measures beyond the traditional command and control [42]. The de-centered approach thus basically allows non-state actors to be involved in producing, monitoring and enforcing regulations that are aimed to influence behavior and regulatory outcomes. In the case of substandard case management among PPs, health agencies could thus collaborate with the key stakeholders (e.g. registration body, licensing body, medical association, patient group, civil societies and representatives of PPs) to set case management standards and establish information exchange mechanisms [37]. The emphasis on co-regulation could facilitate negotiated agreement on roles and responsibilities between the PPs and other actors, leading to a new role and capacity for health authorities, requiring engagement and collaborative forums [37].

## Discussions

Expecting that NTPs could collaborate effectively with thousands of private providers in the context of limited resources and competing resources has proven to be unrealistic. The longstanding dismissal of the essential role of regulation has thus contributed to persistent inappropriate TB case management practices among PPs, with dire consequences for NTPs and patients. Regulation clearly has its challenges and cannot be expected to solve all the problems associated with PPs (as there is no silver bullet to resolve any complex challenge). Nonetheless, governments must not shirk from the challenges of regulatory responsibilities as it is their duty to act in the best interests of the whole population [43]. Poor regulation of health care is a symptom of poor governance [35]. The lack of regulation thus actually sends the wrong messages about acceptable case management practices among PPs.

The non-conventional regulatory strategies and approaches described here are not intended to provide a one-size-fits-all solution to poor case management practices among PPs in LMICs. Regulatory systems in these countries operate in the context of different political conditions and face different sets of challenges. Hence, the presented regulation strategies and approaches will have to be adapted to suit the particular contexts, needs and availability of resources. It is also recognized that regulatory approaches are more likely to succeed if packaged in bundles [44]. Notably, there is no evidence yet that the non-conventional strategies and approaches described above will work in addressing the problem of poor case management practices among PPs in LMICs. That remains to be tested. The limited evidence of effectiveness actually applies to the general scope of health care regulatory approaches in LMICs [37]. This is mainly due to the lack of opportunities to test regulatory interventions, which in turn block much needed progress. Fundamentally, regulation should not be static but should rather be continuously adapted [44]. Thus regulations should always be introduced along with rigorous analytic approaches [44].

Efforts to assist LMICs in the development of regulatory frameworks and techniques unfortunately are still lagging [37]. There is also a lack of efforts in institutional capacity and seeking different ways of achieving regulatory objectives. Regulation clearly needs adequate resources in order to be successfully implemented. Investment requirements to strengthen local capacity for health care regulation could be relatively high, but it will help countries secure their ability in the long run to address the challenges raised by the rapid growth of private health providers in resource constrained settings [45, 46], independent of donor support. Thus it offers a potentially sustainable solution to a longstanding problem. Clearly, effective TB control in LMICs requires effective regulation of TB case management among PPs. Multilateral funding agencies (e.g. the Global fund to Fight AIDS, TB and Malaria) and bilateral funding agencies (e.g. USAID) committed to TB control should provide investment for piloting innovative regulatory strategies and strengthening goverment’s capacity to effectively regulate private care providers in LMICs.
